# Barriers and facilitators to older patients’ counselling in community pharmacy, perceived by pharmacy professionals – a focus group study

**DOI:** 10.1186/s12877-026-07785-0

**Published:** 2026-07-04

**Authors:** Rita Pedro, Rui Resende, Ana Reis, Ramona Mateos-Campos, Agostinho Cruz

**Affiliations:** 1 REQUIMTE/LAQV, ESS, Polytechnic of Porto, Rua Dr. António Bernardino de Almeida, Porto, 4200-072 Portugal; 2 Faculty of Pharmacy, University of Salamanca, Campus Miguel de Unamuno, C. Lic. Méndez Nieto, s/n, Salamanca, 37007 Spain; 3 Escola Superior de Desporto e Lazer, SPRINT - Sport, Physical Activity and Health Research & Innovation Center – (Centro de investigação & inovação do desporto, atividade física e saúde), Instituto Politécnico de Viana do Castelo, R. Dom Moisés Alves Pinho 4900, Viana do Castelo, 4910-023 Portugal

**Keywords:** Barriers, Facilitators, Community pharmacy, Older patients

## Abstract

**Background:**

The ageing of world population is simultaneously a great triumph and a great challenge. It is crucial to adapt health care systems to older population and community pharmacies are not an exception.

**Aim:**

This study aims to identify barriers and facilitators perceived by pharmacy professionals, that may influence older patients’ counselling in community pharmacy.

**Method:**

Four focus groups were conducted with 18 pharmacy professionals with at least three years of professional experience in community pharmacy and who were currently working in community pharmacies in Portugal. The meetings were held via Zoom. The audio was recorded, the interviews were transcribed *ad verbatim* and the data were managed using NVivo (version 15).

**Results:**

Three main themes emerged: barriers centered in older adults, centered in physical space and centered in pharmacy professionals. Twelve categories emerged from the data analysis. Literacy, aisles and access and time management were most mentioned categories of each theme, respectively. Also, several facilitators were identified, which were organized according to the same three themes, with an additional one, facilitators centered in society. Ten categories were identified. Human skills, sitting attendance, skills and management of prescriptions were the most cited ones of each theme, respectively.

**Conclusion:**

This study demonstrate that it is urgent to involve community pharmacies into age-friendly initiatives, to create safe environments for older population which is one of the most frequent patients in community pharmacies.

**Trial registration:**

The project was approved by the Ethics Committee of School of Health of Polytechnic of Porto (reference number CE0084B).

## Background

The ageing of world population is simultaneously a great triumph and a great challenge [1]. According to Eurostat, in 2024, 24.1% of Portuguese population had 65 years and more, a higher number than the provisional percentage of European Union (EU) for the same year (21.6%) [2]. In 2023, EU has a life expectancy at age 65 estimated at 20.2 years. Portugal is above with life expectancy at age 65 of 21.1 years [3]. Life expectancy in EU has been increasing but knowing if the increase of longevity is in a good or bad health is a crucial question. To answer this, Eurostat adopted a statistical indicator named “healthy life years” also called “disability-free life expectancy” which is defined as “the number of years that a person is expected to continue to live in a healthy condition (i.e. without any activity limitation)”. In 2022, in EU, women at age 65 had 9.2 of healthy life years, whereas men had 8.9 years. In Portugal, women at age 65 are expected to have 7.3 years of health life, whereas men have 8.6 years [4].

To create and to adopt measures to help older population remain healthy and active is mandatory. It is crucial to adapt health care systems to older population and community pharmacies are not an exception. Older adults have unique needs not only because of the necessity of multiple medications to delay and treat chronic diseases, but also because of age-related physiological changes.

The role of community pharmacists has become more important throughout the years not only because of the increasing responsibilities, but also because they are the most accessible health professionals to the public. Since pharmacy professionals are located within community, they have easy access to the patients, being in a privileged position to health promotion activities and to be involved in awareness campaigns [5–7].

So, it is essential to identify barriers that affect proper care given to the older population in community pharmacies, so they can be properly mitigated [7]. This study pretends to explore pharmacy professionals’ perspectives and experiences, regarding older patients’ counselling and attendance. The goal was to identify barriers and facilitators perceived by pharmacy professionals, that may influence older patients’ counselling in community pharmacy.

## Methods

### Study design

To address the main objectives, the investigation team decided to conduct a qualitative study using focus groups, following an inductive analytic approach [8]. A focus group is a qualitative data collection technique that allows the investigator to achieve detailed data from a selected group of participants [9, 10]. The main difference between focus groups and group interviews is that in interviews, the researcher controls the dynamics by asking questions and guiding the dialogue. On the contrary, a focus group has a moderator who acts as a facilitator of the discussion between the participants. In a focus group, the moderator plays a secondary role and helps to explore the selected topics in a structured and organized way [9, 11]. According to Powell and Single (1996, p. 1), “a *focus group is a group of individuals selected and assembled by researchers to discuss and comment on*,* from personal experience*,* the topic that is the subject of the research*”.

Qualitative research provides valuable insights into the reasons behind people’s actions or behaviours, allowing a deeper understanding of their underlying motivations [12]. Among the available methods, focus groups were selected as an appropriate tool to gather the data required for this study. There are various approaches to design a focus group, and in this case a multiple-category design was chosen, as it allows comparisons across different categories [13]. The research design was developed with careful consideration of key factors, including the accessibility of the participants, the relevance of the topic, the ethical standards of data collection, appropriate methods of recording, rigorous data analysis, and the authentic representation of pharmacy professionals [14]. For the purposes of this study, older adults are defined as individuals aged 65 years or older [15, 16].

### Participants

The inclusion criteria were pharmacy professionals with at least three years of professional experience in community pharmacy and who were currently working in community pharmacies in Portugal. The recruitment was conducted through social media platforms, such as Facebook, Instagram and LinkedIn, using a visual announcement that included a brief explanation of the sessions, the main objectives, the registration form, and the contact details of the main investigator for any inquires. The registration form requested the participant’s name, years of professional experience in community pharmacy, and the location of their current pharmacy.

### Focus groups design

To ensure the sessions were developed and conducted in a structured manner, a script was prepared. The script was designed taking into account the following: short and clear questions, language that is understood by the target population, and open-ended questions that promote conversation and discussion about the topics [13].

The script consisted of six questions: an opening question, which was simple and direct to encourage participation *(“How long have you been working in community pharmacy*,* and in how many pharmacies have you worked?”)*, a transition question *(“Concerning the older population*,* what conditions or particular characteristics do you identify that require specialized care?”)* and key questions, such as *“What physical barriers do you believe patients face in the pharmacy*,* and how do you think the pharmacy minimizes or eliminates them?”* or *“ If you had decision-making power*,* what changes would you make*,* or what would you do differently in the pharmacy where you work?”*. Participants were encouraged to share their perceptions freely, regarding their professional experience in community pharmacy. The script was made according to the available time and formulated in language appropriate to the target population and designed to foster a non-threatening and permissive environment [11, 13].

The script was validated by an expert in this methodology from Social and Human Sciences. A pilot focus group was also conducted to verify if the written script was correctly designed to generate the intended type of discussion. Since the goals were met, no modifications were deemed necessary.

### Data collection

A total of four focus groups were conducted: three consisting of four participants each, and a fourth comprising six participants. Overall, 18 pharmacy professionals participated. Data from the pilot group were excluded from the analysis. The participant’s number was assigned based on their focus group and participant number. For instance, *participant #3.4* refers to the fourth participant in focus group three.

Each session lasted approximately 90 min, and the audio was recorded to allow later transcription and data analysis. Audio recordings were stored on a memory card under the exclusive custody of the main investigator. All the participants have signed an informed consent. All participant identification data remained strictly confidential to ensure total anonymity throughout the study.

The meetings were held via Zoom – a videoconference platform - to facilitate the participation of pharmacy professionals. The sessions took place between February 2022 and June 2023. The recruitment posed some challenges, as it coincided with the fifth wave of COVID-19 (Coronavirus disease 2019), which placed significant pressure on community pharmacies due to the increased demand for rapid testing. Consequently, professionals were often overwhelmed.

The interviews were transcribed *ad verbatim* and the data were managed using NVivo (version 15), a qualitative data analytics software that supports the organization and exploration of data.

NVivo increases the analysis precision and contributes to a more transparent and reliable qualitative investigation. The software allows the storage of high quantities of data and the correlation between the data could be explored by using mental maps, coding matrix or word frequency [17, 18]. In this study, it was used the coding matrix to analyse the intervention of the participants in each theme and category and to verify if there were discrepancies in codification. Also, it was created mental maps to give a better general view of the identified themes and categories.

### Analysis

The analysis was conducted following the thematic analysis proposed by Braun and Clarke (2012), which enables the systematic identification, organization and interpretation of patterns [19]. Thematic analysis provides accessibility and flexibility to qualitative research, which ensures the critical robustness in qualitative research [20] and interpreting, and creating after deep and “prolonged data immersion, thoughtfulness and reflection, something that is active and generative” [21]. The categorization technique allows the transformation of data into knowledge [22, 23].

The data analysis followed a technique of qualitative research proposed by Resende (2016). After the transcription *ad verbatim*, initial thematic coding was performed, based on the main themes of the script. During this process, segments of the transcripts (units of reference) were grouped into categories based on shared themes. Following the initial coding, the research team reviewed, renamed and refined the categories. In the third phase of the process, all units of reference were reread within each category to refine the process and assure adequacy [24]. This process was led by the principal investigator (a pharmacy professional) under the supervision of an experienced qualitative researcher. Finally, to ensure rigor, the categories were peer-reviewed by a critical friend. A critical friend is defined as “*a trusted person who asks provocative questions*,* provides data to be examined through another lens*,* and offers critiques of a person’s work as a friend*” [25]. For this role, a highly experienced pharmacy professional, with more than 15 years of professional experience was invited. This process enabled the data to be examined from a different perspective, ensuring respectful and constructive feedback, and providing suggestions to clarify the results.

After data analysis, a simple translation of the quotes was made by an English translator for the purposes of publication. No study incentive was offered. The project was approved by the Ethics Committee of School of Health of Polytechnic of Porto (reference number CE0084B).

## Results

A total of 18 pharmacy professionals participated in the four focus group discussions. Two of the groups were composed exclusively of women, while the other two included both men and women. The study participants were predominantly female (89%). Furthermore, 39% of the professionals had been working in the same community pharmacy since the beginning of their professional career, and most had seven or more years of experience in community pharmacy.

From the analysis, three main themes emerged: barriers centered in older adults, barriers centered in physical space and barriers centered in pharmacy professionals. In addition, several facilitators were identified, which were organized according to the same three themes, with an additional one, facilitators centered in society.

### Barriers

**Fig. 1:**
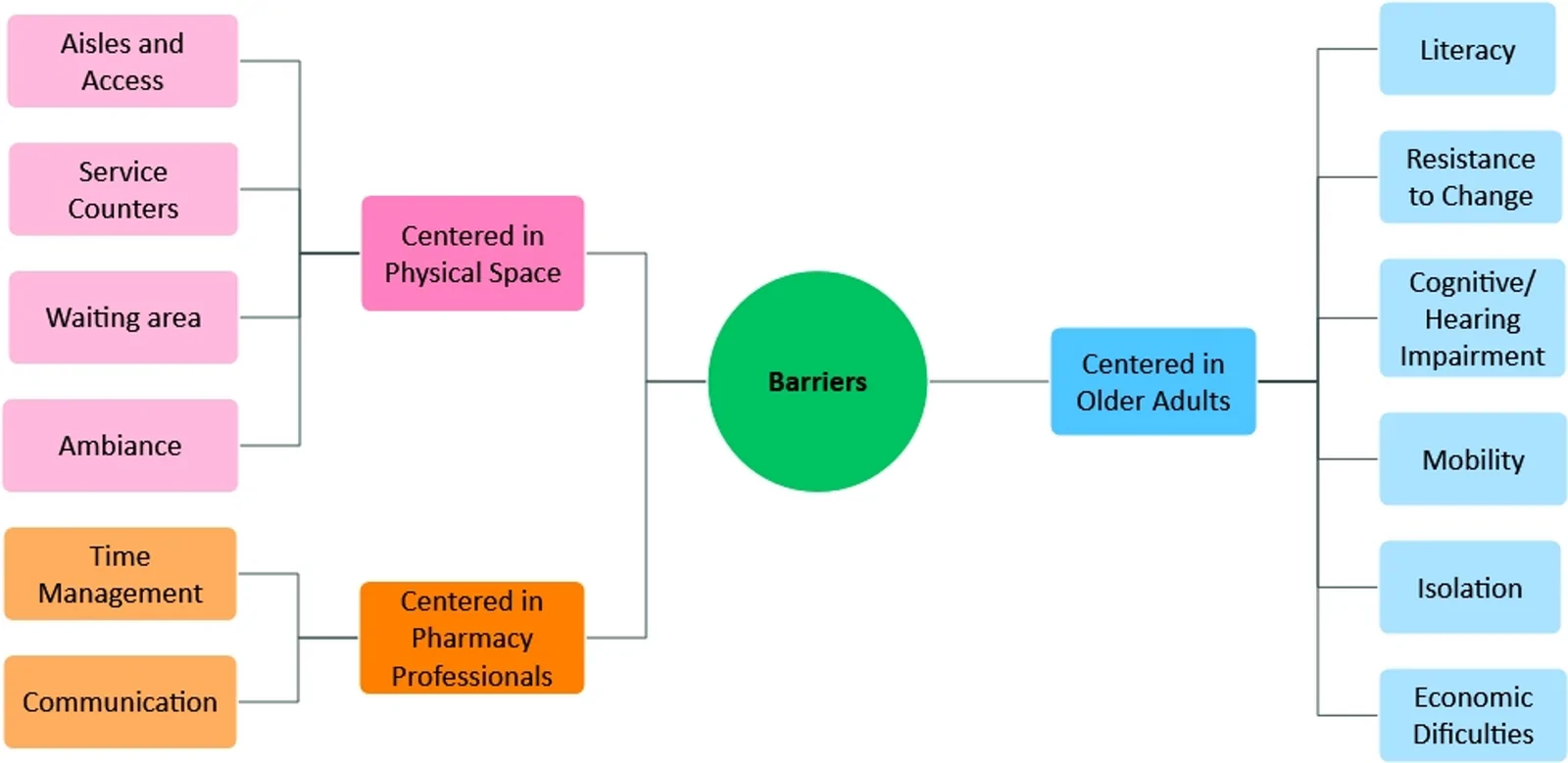
Mental map of barriers - overview of the resulting themes and categories

Regarding the identified barriers, three themes and twelve categories emerged from the data analysis, which are represented in the mental map (Fig. 1).

The coding matrix (Table 1) contains the themes and the total number of units of reference (represented in bold) and the categories with the corresponding number of units of reference for each group.

**Table 1:** Coding matrix of barriers: overview of the resulting themes and categories that emerged from focus groups

	FG2	FG3	FG4	FG5	Total
**Centered in Older Adults**	**28**	**21**	**11**	**16**	**76**
Literacy	12	5	4	8	29
Resistance to Change	7	3	0	5	15
Cognitive/Hearing Impairment	3	6	3	2	14
Mobility	1	4	2	0	7
Isolation	3	3	0	1	7
Economic Difficulties	2	0	2	0	4
**Centered in Physical Space**	**17**	**23**	**13**	**11**	**64**
Aisles and Access	7	13	3	4	27
Service counters	6	3	6	5	20
Waiting area	3	3	1	2	9
Ambiance	1	4	3	0	8
**Centered in Pharmacy Professionals**	**2**	**6**	**4**	**0**	**12**
Time Management	2	4	3	0	9
Communication	0	2	1	0	3

*FG* Focus group

total number of units of reference (represented in bold)

#### Barriers centered in older adults

Barriers centered in older adults were the most represented, comprising six categories: (1) literacy; (2) resistance to change; (3) cognitive/hearing impairment; (4) mobility; (5) isolation and (6) economic difficulties. These categories reflect the complexity of older adults and the aging process itself.

#### Literacy

This was the most mentioned category in this theme. Pharmacy professionals reported that older patients’ knowledge about medications is not always accurate. A lack of understanding regarding medication indications and dosing schedules was highlighted as a significant problem. In addition, digital literacy emerged as a concern, since some older adults experience difficulties managing electronic medical prescriptions that are sent to their mobile phones. Also, although the percentage of illiterate persons has been decreasing, some patients are illiterate.

Their knowledge about their medications is not always accurate. They can easily identify a medicine by its packaging, but if asked which medication they are taking- for example, during an appointment with the doctor or with us - they do not know. (Participant #2.4)

The fact that prescriptions are also sent to mobile phones sometimes makes things a bit difficult, because some patients do not know how to use their phones or read the messages, so they ask us to check for them. I think they feel lost without having the prescription on paper. (Participant #2.5)

There are older adults who, unfortunately, are illiterate and can’t even write. (Participant #4.7).

#### Resistance to chance

Resistance to chance was another category identified in relation to older patients. Such resistance can be about medications, including the use of generics, among others. Furthermore, some older patients fear losing their autonomy, for example when a pharmaceutical service is proposed. In general, pharmacy professionals perceived older adults as being resistant to innovations.

They are people who usually have great difficulty changing their habits; they are normally familiar with a particular medicine box or a specific manufacturer. (Participant #2.4)

I think elderly patients are afraid of losing their autonomy, they feel like they are losing ground in their own lives. (Participant #3.5)

There is also some resistance. If I could change patients’ mindsets regarding letting the pharmacy handle their medication preparation, that would be ideal! It would prevent anxiety between both the patients and their families by preventing medication errors, duplications, and missed doses. (Participant #5.6)

#### Cognitive/Hearing impairment

This category was specifically related to COVID-19 and the use of protective equipment like masks. It is important to remember that these focus groups were conducted during the COVID-19 pandemic - World Health Organization (WHO) declared the end of the state of emergency on May 5, 2023 - so the impact of COVID-19 was very evident. It became clear that these barriers affected hearing and communication.

The loss of their abilities is also noticeable. With the reality of mask-wearing, the fact that they cannot read our lips - especially those who are hard of hearing - makes things a bit complicated. (Participant #2.5)

I believe that, especially now, after COVID, things have become significantly more complicated. Most older adults were isolated for a long time, and there has been a significant decline in their cognitive function. (Participant #3.6)

I believe that physical barriers ultimately created greater difficulties in terms of hearing and communication. (Participant #3.4)

#### Mobility

Some participants highlighted mobility as a barrier: “*The mobility of older patients is always more limited. One way or another*,* they do not have the physical dexterity of an adult*” (Participant #3.6) and “*Some people have great difficulty traveling to the pharmacy*,* and this is particularly noticeable when a product is out of stock and we tell them it will only arrive the next day. Many users report that they simply cannot make the trip or that it is inconvenient*” (Participant #2.4).

#### Isolation

Isolation was identified by participants as a growing problem with increasing impact in the lives of older adults. Due to this, older adults frequently go to community pharmacy to have some extra attention and to socialize.

Many times, older adults, because they feel very lonely, go to the pharmacy to buy medicines and then stay a little longer to talk and make conversation. (Participant #2.5)

Sometimes they aren’t even looking for specific advice; they just want to come to community pharmacy to talk because they are lonely. (Participant #5.10)

#### Economic difficulties

Portugal has been facing some economic challenges, and the population in general - older adults in particular -, has faced difficulties due to the increasing cost of life and the stagnant wages.

The lack of money is also still evident… (Participant #2.7).

Patients struggle to understand why the price listed on the prescription differs from the actual laboratory price. They cannot understand why prices vary so much between different laboratories if the product is supposed to be the same… (Participant #4.3).

### Barriers centered in physical space

Following the barriers centered in older adults, barriers centered in physical space emerged as the most frequently mentioned theme. This theme comprised four categories: (1) aisles and access; (2) service counters; (3) waiting area and (4) ambiance. These categories reflect the range and diversity of different aspects in the physical environment of a community pharmacy.

#### Aisles and access

This was the most prominent category within the theme of barriers centered in physical space. It included aspects such as stairs, access ramps, automatic doors and the arrangement of shelves and aisles.

In the pharmacy where I work, you need to go upstairs to enter. For older adults, this is sometimes a bit complicated. We do have a ramp, but it is not very user-friendly or frequently used. It has a door that must be closed, which is especially difficult for those who use crutches or have trouble walking. (Participant #2.5)

In the pharmacy, it is complicated because it is increasingly necessary to display products and focus on marketing with gondolas, advertisements, and other promotional materials to reach a specific target audience. For customers in general, it is relatively easy, and they even like it because they can look at the products. But for older adults, it is clearly more difficult: they bump into the gondolas, lean on them, and they are not safe! (Participant #3.6)

In the pharmacy where I work, we have an automatic glass door, and we had to put a large red strip on it so that people wouldn’t bump into the door! (Participant #3.5)

#### Service counters

This category was frequently referenced by participants, often in connection with the COVID-19 pandemic. The use of acrylic barriers at pharmacy reception desks was reported to affect both communication and overall comfort, as they reduced available space for personal items such bags or wallets. It was also mentioned the products exposed in reception desks, which also influence general comfort of patients.

The acrylic screens took away the space where they used to lean for support. (Participant #2.4)

COVID made many things more difficult: the PVC, the acrylic screens, the masks — they all became barriers. It was a terrible time. (Participant #4.7)

We kept the acrylic screens to protect the pharmacy staff. Even though masks are no longer mandatory, the acrylic screens themselves still create a communication barrier. However, it’s mainly at the end of the day, when the acrylic is cleaned, that we realize how important they are: it’s a matter of hygiene. (Participant #4.6)

In some cases, service counters are simply small, but for the most part, it’s because they are used, sometimes excessively, for product displays and advertising. It isn’t comfortable to attend under these conditions; it feels like the trust between us and the patient is slightly compromised. They want to be able to place their belongings on the counter, but they can’t. And this is a population that easily gets frustrated when the interaction doesn’t flow smoothly. (Participant #5.8)

#### Waiting area

The existence of chairs in the waiting area was highlighted as particularly important, especially for older patients with reduced mobility, since the waiting times in pharmacies can be long.

There is a problem that arose from COVID, related to the places where patients can sit. Now we only have a single chair in the waiting area. (Participant #2.7)

Even though we have space in the waiting area, we had to reduce the number of seats due to COVID: now we have only one chair. Despite the limits on how many people could be inside the pharmacy, we can’t exactly ask an 80 or 90-year-old patient to wait outside. We ended up bringing out extra chairs so they could wait in a bit more comfort. (Participant #3.5)

#### Ambiance

General noises, such as music playing on the radio or electronic ticket dispensers, were identified as barriers for older patients.

We also have an electronic ticket dispenser, and I feel that older adults get a bit confused about which type of ticket they should get. (Participant #3.4)

We need to pay attention to this: communication is sometimes a barrier. The masks, the acrylic barriers, the nearby counters with other people also talking, the background noise, the radio, the air conditioning… There are many factors that can influence the relationship between us and the patients. These end up being somewhat negative aspects. (Participant #3.3)

### Barriers centered in pharmacy professionals

Finally, the least mentioned theme was related to pharmacy professionals, comprising two categories: (1) time management and (2) communication.

#### Time management

Time management was the most frequently mentioned category, highlighting the difficulties pharmacy professionals face in balancing the need to talk with patients and the pressure of attendance, especially when many people are waiting.

Another negative point is that sometimes older adults need more time during the service, and unfortunately, this is not always possible. As pharmacy professionals, we see the pharmacy filling up with people, and although we need to explain everything calmly to that patient, seeing others in line becoming impatient makes us feel nervous. (Participant #4.6)

Sometimes it’s difficult; it depends on the time of day. For example, when there are a lot of people waiting in the pharmacy, we know the person wants to talk, but we just can’t. We have to attend them and move on to the next patient. It’s a bit hard because we don’t want to dismiss them. (Participant #2.5)

#### Communication

The way pharmacy professionals communicate with older people was also mentioned as a potential barrier, for example “I think that sometimes what happens is that older adults are infantilized in the pharmacy… I have complaints from patients who say that it feels as if they are no longer worth anything or as if they are no longer able to understand.” (Participant #4.4). Also, the language spoken by the patient, naturally influences the success of the communication, “One of them is Indian and he always ends up with me because I’m the one who speaks English best, and that’s also a barrier.” (Participant #3.3).

### Facilitators

**Fig. 2:**
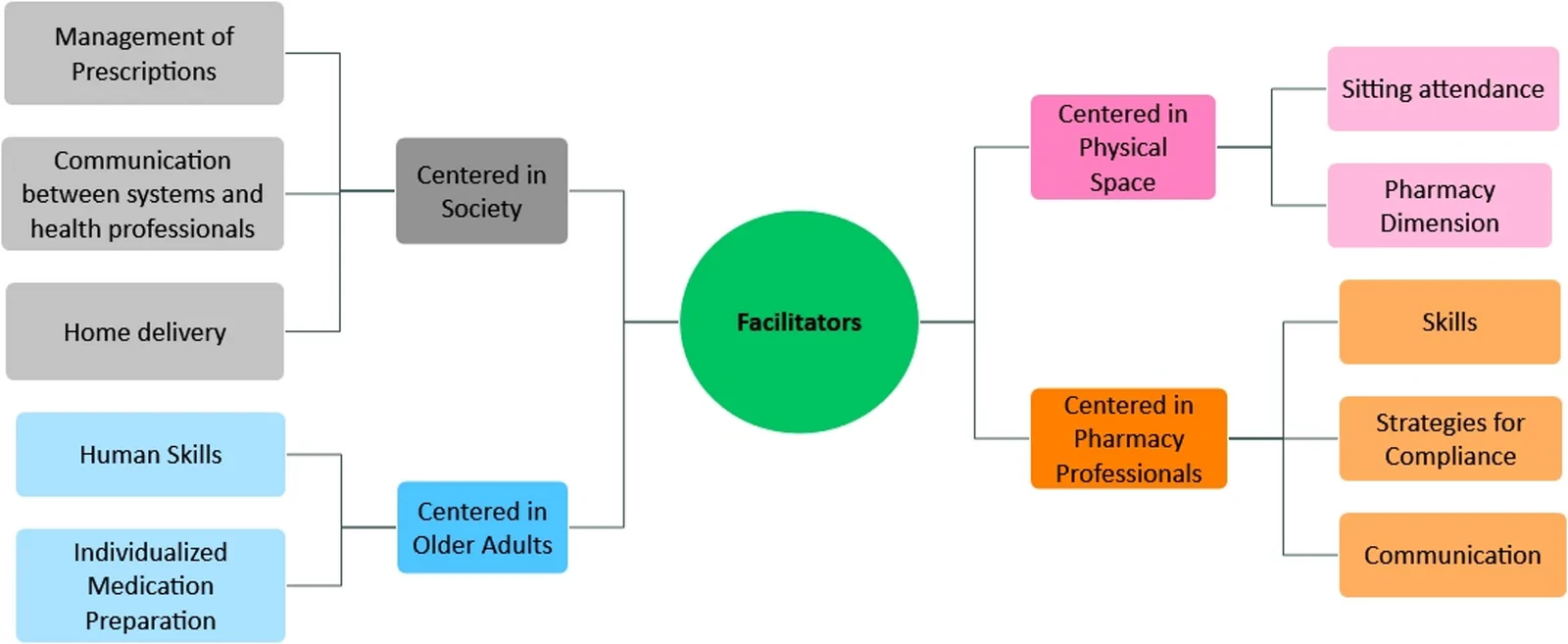
Mental map of facilitators - overview of the resulting themes and categories

During the data analysis, several facilitators were also identified and categorized using the same process. Four themes and ten categories were identified, which are represented in the mental map (Fig. 2).

The coding matrix (Table 2) contains the themes and the total number of units of reference (represented in bold) and the categories with the corresponding number of units of reference for each group.

**Table 2:** Coding Matrix of Facilitators: overview of the resulting themes and categories that emerged from focus groups, with the total number of units of reference for each group

	FG2	FG3	FG4	FG5	Total
**Centered in Pharmacy Professionals**	**19**	**22**	**12**	**15**	**68**
Skills	5	12	5	4	26
Strategies for compliance	7	6	4	7	24
Communication	7	4	3	4	18
**Centered in Society**	**15**	**8**	**8**	**8**	**39**
Management of prescriptions	12	2	8	2	24
Communication between Systems and Health Professionals	2	5	0	5	12
Home delivery	1	1	0	1	3
**Centered in Older Adults**	**8**	**10**	**1**	**10**	**29**
Human Skills	5	6	1	6	18
Individualized Medication Preparation	3	4	0	4	11
**Centered in Physical Space**	**1**	**7**	**1**	**4**	**13**
Sitting Attendance	1	5	1	4	11
Pharmacy dimension	0	2	0	0	2

*FG* Focus group

total number of units of reference (represented in bold)

### Facilitators centered in Pharmacy Professionals

This was the most mentioned theme and comprises three categories: (1) skills; (2) strategies for compliance and (3) communication.

#### Skills

This category referred to the skills of pharmacy professionals and encompassed characteristics such as resilience, adaptability, patience, the ability to repeat information multiple times, caring attitude, active listening, empathy, and knowledge.

I think that most of our life in community pharmacy is, undoubtedly, about adjustment, adaptation, and resilience. (Participant #2.7)

Being patient, explaining more than once, paying attention to the patient, and showing caring. (Participant #2.5)

#### Strategies for compliance

Several strategies to promote compliance were mentioned during the focus groups. These included practices such as cutting out the name of an old medication and attaching it in the new one, using tags, stickers, pictograms, using different colours, printing digital prescriptions, repeating instructions and advice, and asking patients for feedback to ensure that they understand every explanation. In addition, pharmaceutical consultations were highlighted to monitor patient adherence.

Older adults are very visual. Saying something different can sometimes complicate things. Colours make it easier, so sometimes we remove the name or manufacturer from the old box and paste it onto the new one to simplify things for them. (Participant #2.6)

The labels available in the software for printing are excellent, because many times patients could not clearly read our handwriting (when we wrote on the boxes). And the pictograms showing times of the day help a lot, as they are more concise and clearer—anyone can understand them. (Participant #2.7)

#### Communication

Communication can act both as a barrier, as previously mentioned, and as a facilitator. When pharmacy professionals use a simple and clear language, short sentences adjusted to the patient and an appropriate voice projection, communication can positively influence the success of the attendance.

We need to use simple language and provide clear and concise information. (Participant #2.7)

I believe it’s important to project your voice clearly to make understanding easier. Since many older adults’ experience hearing difficulties, it’s essential not to speak too softly and to raise your voice slightly. Speaking more slowly and paying close attention to the words we choose helps prevent them from feeling embarrassed about not understanding or hearing, which might cause them to ask again. (Participant #4.6)

### Facilitators centered in Society

During data analysis, a fourth theme emerged - facilitators centered in society - which comprises three categories: (1) management of prescriptions; (2) communication between systems and health professionals and (3) home delivery.

#### Management of prescriptions

The most frequently cited category was the management of prescriptions. Participants highlighted facilitators such as the automatic revalidation of chronic medication by pharmacy professionals, which can reduce suspended sales and waiting lists in primary care. In addition, some patients reported that scheduling a medical appointment is sometimes more expensive than paying for the medication without the government co-payment. The suspended sales were described as a real problem that influences, among other things, customer loyalty to community pharmacies.

I think it would be excellent if pharmacy professionals could carry out the revalidation of chronic medication. (Participant #2.7)

Some patients say they do not want to spend money on a medical appointment just to request a medication, especially when the medication itself is cheaper than the appointment. (Participant #2.5)

We can be firm and refuse to dispense without a prescription, but if patients go to the pharmacy next door and they dispense it, it becomes very complicated. Our policy is to look after the patient, and we try to be flexible in supporting them. (Participant #2.7)

#### Communication between systems and health professionals

This category was another frequently mentioned facilitator. Participants suggested that greater integration of informatic systems across hospitals, community pharmacies, private practices, and health centres, would be truly advantageous - not only for patients, but also for the national health system. Improved communication could help reduce the number of medical appointments needed solely for prescription renewals, as well as costs associated with avoidable hospital admissions.

The idea is that both the doctors’ system and ours should be connected. The doctor makes the virtual prescription, and we carry out the validation. In the case of chronic medication, pharmacy professionals could request the renewal, thus avoiding the need for the patient to go to the doctor. If we detected a prescription error, we would be able to quickly inform and clarify it with the doctor, without any problem. (Participant #2.4)

I would have pharmacy staff working with doctors in health centers. There’s a massive communication failure between doctors and patients, which patients often try to make up for at the pharmacy. It would likely reduce some medical errors, but mainly, it would just make communication much easier. (Participant #3.6)

I would add that having more clinical knowledge would be advantageous—specifically, access to each patient’s routine medication record. Patients often can’t accurately say what medication they take regularly. When we want to provide advice for a specific situation, we often feel hesitant because we don’t know what they are actually taking. I believe that knowing their therapeutic regimen would give us greater confidence during attendance. (Participant #5.8)

#### Home delivery

Although home delivery was a service prior to COVID-19, the pandemic considerably amplified its role, transforming a pre-existing trend into a strong facilitator:

Regarding deliveries, it was something we were already doing before, but during the pandemic, we began doing much more home delivery. There were situations where elderly users would visit the pharmacy themselves but would later ask for their medications to be delivered to their homes—for instance, if an item was out of stock or had been forgotten. (Participant #3.3)

It often happens that, because this is a relatively small community, if a patient can’t make it to the pharmacy, we take the medication to their home. There are a shared connection and a personal knowledge of the patient that allows us to provide better care. (Participant #5.6)

### Facilitators centered in older adults

Facilitators centered in older adults was one of the least represented themes, comprising two categories: (1) human skills and (2) individualized medication preparation.

#### Human skills

This category encompasses several characteristics of older patients that pharmacy professionals recognized as facilitators in their interactions, including loyalty, trust, time and patience.

Older adults are usually much more loyal to the community pharmacy… They are always the same faces, and they consistently seek the support and advice of the professionals who work there. (Participant #2.4)

They have an enormous trust in us, because they receive support and guidance that they often do not ask their doctors for. (Participant #2.7)

Time and patience. While other people come in a hurry and in a rush, with older adults we can provide care more calmly, explain things properly, and not feel pressured. (Participant #4.4)

#### Individualized medication preparation

Pharmacy professionals considered individualized medication preparation to be a pharmaceutical service of added value for the older population, particularly in the context of polypharmacy and in helping to prevent medication lapses.

We have been trying to implement the Individualized Medication Preparation Plan to make it easier for them, so they know what type of medication they need to take and at what time… Therefore, individualized medication preparation has become a real advantage for this population. (Participant #2.7)

At the rural pharmacy where I worked, we offered Individualized Medication Preparation, and it was well-received by older patients, essentially the ones that are taking 5 to 6 pills a day. Often, we were initially approached by their caregivers, who wanted to understand the service before trying to convince their parents to use it. (Participant #3.4)

### Facilitators centered in physical space

Facilitators centered in physical space were the least representative theme and fell into two categories: (1) sitting attendance and (2) pharmacy dimension.

#### Sitting attendance

Although most pharmacies don’t have a designated space for sitting attendance, sometimes it is necessary to improvise. Many pharmacy professionals mentioned that they sometimes need to attend with the patient seated in the waiting area or by placing a chair in front of the attendance desk because some older patients can’t stand for long periods. Some community pharmacies already have sitting attendance integrated with a pharmaceutical service – pharmacotherapeutic follow-up.

It has also happened that we had to place a chair with the patient sitting in front of us at the counter. Older adults sometimes have difficulty waiting, so we try to create conditions to provide them some comfort. (Participant #3.5)

I know of another pharmacy in the area that has a seated counter, where both the pharmacy professional and the patient sit, and those appointments can last about an hour! But that pharmacy offers a pharmacotherapy follow-up service, which is why they have a seated counter. Naturally, the patient pays for that service. (Participant #3.6)

#### Pharmacy dimension

Having a spacious physical environment was also identified as a facilitator related to the physical space.

Physically, pharmacies should have a more spacious venue with better access. (Participant #3.6)

Currently, the pharmacy where I work is quite large and has no steps, which is important for our patients. (Participant #3.4)

### Thematic overview

Table 3 shows an overview of the identified barriers and their corresponding facilitator.

**Table 3:** Overview of the identified barriers and facilitators that may overcome these barriers

Barriers	Facilitators that may overcome the identified Barriers
*Centered in Older Adults*	
Literacy	Strategies for Compliance and Communication (*Centered in Pharmacy Professionals*)
Resistance to Change	Strategies for Compliance and Communication (*Centered in Pharmacy Professionals*)
Cognitive/Hearing Impairment	Communication (*Centered in Pharmacy Professionals*)
Mobility	Sitting attendance and pharmacy dimension (*Centered in Physical Space*)
Isolation	Home delivery (*Centered in Society*) and Individual Medication preparation (*Centered in older Adults*)
Economic Difficulties	Non defined
*Centered in Physical Space*	
Aisles and Access	Pharmacy dimension (*Centered in Physical Space*)
Service counters	Non defined
Waiting area	Sitting attendance (*Centered in Physical Space*)
Ambiance	Non defined
*Centered in Pharmacy Professionals*	
Time Management	Non defined
Communication	Human skills (*Centered in older Adults*)

## Discussion

In this study it is clear the differences between the type of barriers mentioned. The most mentioned ones were barriers centered in older adults, englobing variables like literacy, resistance to change, cognitive/hearing impairment, mobility, isolation and economic difficulties.

The most mentioned category of this study was literacy. A recent cross-sectional study that aimed to investigate the levels of health literacy demonstrated by older adults in Portugal conclude that 80.6% of respondents (total of 613 participants) revealed limited general health literacy [26]. Also, there are already established health literacy guidelines that provide clinicians with standardized frameworks for effective communication. These tools allow providers to tailor their counseling to meet individual patient needs, with particular emphasis on supporting older adults [27, 28].

Resistance to change, as well as cognitive and hearing impairments, are common manifestations of normal aging characterized by functional decline. For instance, reduced dopamine levels have been linked to increased cognitive rigidity and resistance to change [29]. These age-related impairments are well-documented in the literature as significant barriers to therapeutic adherence, often leading to poor compliance when medical instructions are not fully heard or understood [30–32].

Another mentioned barrier was mobility which can be justified by muscle failure, known as sarcopenia, a common pathology among adults of older age [33].

Isolation was also a category that emerged from this study and unfortunately is not new. The WHO Commission on Social Connection recently released a global report revealing that loneliness and isolation affects nearly one in six people globally [34]. Isolation was also a self-perceived barrier that could explain the frequent community pharmacy visits of older patients [32].

Finally, the least mentioned category related to older adults was economic difficulties. This is a significant concern, particularly in the context of raising inflation and cost of living. In 2023, Portuguese poverty rate was more than 20% for the elderly [35].

Barriers centered in physical space emerged as the second most frequent theme. These findings align with established literature, reinforcing a well-documented challenge within the field of health aging. In 2007, WHO published a guide for Global age-friendly cities with the definition of eight topic areas, such as outdoor spaces and building, community support and health service and communication and information. Many themes are mentioned in the guide, but nothing is referred related to community pharmacy environment. Which is truly contradictory since it is mentioned health services who prevent disease and promote health [36]. Also, the guide for global age-friendly cities refers that building and housing design should be barrier-free [36]. Although this guide does not mention pharmacy settings – which is a significant gap – the pharmacy layout should also take this into account. Aisles and access, service counters and waiting area were barriers identified by pharmacy professionals in this study. Coincidentally, were also barriers identified by older adults in a similar study, which is very curious and should be considered since physical space can directly influence the success of communication and the information given [32, 37, 38].

Ambiance was another identified barrier englobing variables like general noises, radio music and electronic ticket dispensers which aligns with published literature that highlights the importance of the pharmacy design. Pharmacy users affirmed that their level of comfort could be affected by the size, structure and design of the pharmacy space [38].

The least mentioned theme were barriers centered in pharmacy professionals. The limited number of references in this theme may be explained, hypothetically, by the fact that the focus groups were conducted with pharmacy professionals themselves. As a result, participants may have found it more difficult to identify weaknesses in their own practice. Time management was the most cited category. The role of community pharmacists is expanding globally so is their functions and responsibilities. Managing time during the daily practice is a real challenge. A recent study affirms that 60% of Portuguese community pharmacists works 40 to 45 h per week, which is above the median week hours of Portuguese population (39.9 h) [39]. Also, an Australian study affirms that pharmacists were busy, undertaking on average 25 tasks per hour [40]. Another cited barrier was communication. The way pharmacy professionals communicate is crucial to therapeutical success and therapeutical adherence. Portuguese health authorities published a good practice manual for health literacy that identify specific opportunities for community pharmacies, such as detecting non perceived patient necessities, minimize errors by giving total knowledge about therapeutic regimen and to constantly promoting health literacy [27]. Patient-centered care communication is considered an essential element to build a solid and appropriate relationship with the patient [41].

Identifying barriers is essential, but the real priority lies in establishing ways to overcome them. In this study, it was possible to associate some facilitators to the identified barriers. For example, to combat lack of mobility in older adults, having a sitting attendance and a pharmacy with an adequate dimension could help. Resistance to change and low literacy were identified barriers centered in older adults and could be overcome with the use of strategies for compliance and an adequate communication. Adequate communication can also help in cases of cognitive or hearing impairment. Another example is home delivery and individualized medication preparation that could be good strategies in cases of older person’ isolation. Regarding physical space, pharmacy dimension and sitting attendance may mitigate the barriers related to aisles and access and related to waiting area, respectively. Concerning pharmacy professionals, human skills can overcome the barriers related to communication. It is important to highlight that not all barriers had corresponding facilitators and vice-versa. Most facilitators were mentioned when the main investigator asked, during the focus group, ““*If you had the power to decide*,* what would you do differently at your regular pharmacy?*”. Some of the identified facilitators are not linked to any barrier, which emphasize the need for future studies.

Malet-larrea and colleagues (2019) [42] developed a study that aimed to define and implement the concept of “Age-friendly Pharmacy” which had fifteen criteria clustered into four topics: relationships, pharmacy layout, pharmaceutical services and communication of services. It is curious to attest that some of the barriers and facilitators that were identified by this study are like some criteria for age-friendly pharmacies. For example, regarding relationships, the importance of showing interest and empathy for the consumer’s health problems, the use of appropriate language and tone and to be attentive. About pharmacy layout, the need to transform the space, to eliminate architectural barriers and to have chairs or seats available in waiting area. Also, the huge importance of existing a coordination between community pharmacy and health centres, by having the possibility of contacting the physician by telephone or email [42]. Besides this, the American Society of Consultant Pharmacists, Peter Lamy Center on Drug Therapy and Aging and John A. Hartford Foundation has also defined a strategy for age-friendly pharmacists: the 4Ms framework. This framework is about what matters (to align care with each specific health outcome), medication (the importance of choosing age-friendly medication), mentation (the need to prevent, identify and treat delirium) and mobility (ensure that older adults can move safely) [43]. The proclamation of the Decade of Healthy Ageing (2021–2030) by WHO Member States lead to global efforts to reaffirm the commitment of member states with a healthier ageing. There were many measurement domains and indicators to evaluate the success of actions implementation. One of the four action areas of the decade was “*to deliver person-centred*,* integrated care and primary health services that are responsive to older people*” and yet, the only refence to pharmacies is the pharmacological treatment that an older person diagnosed with depression might have. Contradictorily, geriatric care is defined as a multidisciplinary teams’ approach to caring for older people that may include, among other health professionals, consultant pharmacists [16]. Age-friendly environments must be supported by strategies defined by public authorities to avoid the existence of physical and social barriers. Portugal is already working on age-friendly environments but, in 2018, less than 10% of national Portuguese population was covered by activities related to global network for age-friendly cities and communities [44]. It is urgent that authorities define and implement concrete action plans, with measurable indicators, for a truly active ageing, integrated in communities and societies. There are a lot of strategies and initiatives, but the problems related to ageing are still a reality. This study highlights the need for a checklist or a good practice manual to empower community pharmacy managers in reorganizing physical spaces and professional skills, while addressing identified barriers and facilitators.

### Strengths and limitations

The strength of this study was the input from pharmacy professionals which are involved into daily practice, with high experience in older patients’ attendance and counselling. This study identified barriers and facilitators that adds new knowledge and support the existing literature about age-friendly pharmacies.

A limitation was the reduced number of participants (*n* = 18) which is explained by the recruitment difficulties mentioned above. Even though the number of participants was not high, the research team believe that data saturation was achieved since no new categories emerged after the fourth session, indicating that saturation had been reached.

Other common limitation of focus group methodology is the difficulty to generalise the qualitative data or the eventual subjective interpretation. This limitation was overcome by the involvement of a critical friend. Being an exploratory study, the aim was to develop interpretations that lead to deep insights and studies, rather than generalize findings to similar populations [45]. Future research can overcome this limitation by utilizing larger cohorts to validate these thematic insights at an international level.

Besides this, focus groups were conducted during the COVID-19 pandemic, which may have had impact on some results, influencing the identified barriers and facilitators. This limitation could be overcome by repeating the focus groups, with the same script, and then compare the obtained results.

## Conclusions

While extensive research has addressed age-friendly environments and initiatives, a significant gap remains regarding community pharmacy settings, both in Portugal and internationally. This study fills that gap by providing a comprehensive qualitative analysis of barriers and facilitators according to pharmacy professionals’ perspectives. This study suggests that involving community pharmacies in age-friendly initiatives is essential to creating safe environments for older adults, who represent one of the most frequent patient groups. A checklist with the main facilitators and the barriers that should be avoided would help public authorities and community pharmacies directors adapt and change community pharmacy environments.

## Data Availability

The datasets used and/or analysed during the current study are available from the corresponding author on reasonable request.

## Appendix

### Pharmacy Professionals | Script

#### Introduction (time reference: 10 min)

- Presentation of the main investigator.
- Reading the informed consent.
- Clarification of some points: duration of the session, audio recording, any doubts, ….

#### Opening question (time reference: 1 min/participant)

As you know, what I am proposing today is a discussion to share ideas about community pharmacies and older patients’ counselling. How long have you been working in community pharmacy, and in how many pharmacies have you worked?

#### Transition question (time reference: 1 min/participant)

Thank you for your contributions. I see we have different realities here, which is great. We all acknowledge that the public in a community pharmacy is naturally diverse, with the older population being a very frequent presence. Concerning the older population, what conditions or particular characteristics do you identify that require specialized care?

#### Key questions (time reference: 15 min per question)

1. Thank you for your contributions. Now that you have identified the characteristics of this age group: which aspects arising from the particularities you have just mentioned may positively or negatively influence the quality of service?
2. I would now like to discuss the physical setting of a community pharmacy. The relationship established between the pharmacy professional and the patient, as well as the setting in which they interact, can influence the patient’s well-being and the quality of care. Keeping in mind the characteristics of the older patient you just mentioned, what physical barriers do you believe patients face in the pharmacy, and how do you think the pharmacy minimizes or eliminates them?
3. We have discussed the characteristics of older patients and the physical barriers within the community pharmacy. Let us now focus on our role as healthcare professionals: what characteristics do you believe are necessary for a pharmacy professional to provide high-quality care to an older patient, taking into account the positive and negative aspects mentioned previously?
4. Thank you all for your participation and contribution. I would like to ask you one last question: If you had decision-making power, what changes would you make, or what would you do differently in the pharmacy where you work?

#### Conclusion (10 min)

- Summary of the main ideas.
- Express gratitude for the availability of the participants.

## Abbreviations

COVID-19: Coronavirus disease 2019
EU: European Union
FG: Focus group
WHO: World Health Organization
